# The urban-rural disparity in the prevalence and risk factors of hypertension among the elderly in China—a cross-sectional study

**DOI:** 10.7717/peerj.8015

**Published:** 2019-11-07

**Authors:** Hongxun Song, Da Feng, Ruoxi Wang, Jian Yang, Yuanqing Li, Junliang Gao, Zi Wang, Ziqi Yan, Chengxu Long, Jiawei Zhou, Zhanchun Feng

**Affiliations:** 1 School of Medicine and Health Management, Tongji Medical College, Huazhong University of Science and Technology, Hubei Province, People’s Republic of China, Department of Health Management, Wuhan, Hubei, China; 2 School of Pharmacy, Tongji Medical College, Huazhong University of Science & Technology, Wuhan, Hubei, China

**Keywords:** Prevalence, Hypertension, Urban, Risk factors, Rural, Chinese

## Abstract

**Introduction:**

This study aimed to assess the prevalence of hypertension and to explore the disparities of its risk factors among urban and rural elderly.

**Method:**

Data of hypertensive patients were collected from the China Health and Retirement Longitudinal Study (CHARLS) 2015. Stratified sample households were selected from 450 villages or communities of 150 counties from 28 provinces. Multivariable logistic regression was performed to analyze the factors correlated with hypertension.

**Results:**

Prevalence of HBP was 47.6% (95% CI [45.2%–50.1%]) in total and it was close between urban and rural population (48.6% vs 47.2%). Factors associated with HBP were different between urban and rural areas. In urban areas, hypertension was significantly associated with literacy and diabetes in both genders, high BMI level and smoke quitters in males, and physical activity and dyslipidemia in females. In rural areas, hypertension was significantly associated with older age, higher BMI level in both males and females, and dyslipidemia in males.

**Conclusions:**

The prevalence are about the same among urban and rural residents, but their risk factors vary from each other. Disparity in the risk factors between urban and rural population should be taken into consideration for further intervention.

## Introduction

Hypertension is common in China and its prevalence is rising as the population grows older. More than 1/4 of Chinese were suffering from hypertension since 2012 according to Nutrition and Health Monitoring of Chinese Residents. Most of them were elderly. Overall, 27.8% of Chinese adults were hypertensive, with the prevalence higher in men than in women and increasing steeply with rising age (Li et al., 2017b). In 2013, 31.1% of the world’s adults had hypertension (Mills et al., 2016).

Hypertension is associated with higher risks for cardiovascular disease (CVD) mortality and accounts for about one third of deaths (Lewington et al., 2016). As the main target of intervention to reduce CVD risk event (Bi et al., 2015; Li, Zhao & Hu, 2016; Yan et al., 2014). Individual characteristics, social economic status, behavioral health, and comorbidities were common risk factors for hypertension in urban and rural elderly. (Yin et al., 2016; Zhang, Wang & Joo, 2017). For example, risk of hypertension increases with age and BMI level (Xu, Lai & Fang, 2016). Hypertension is associated with healthy behaviors including smoking, drinking and physical activity. Also, those who had chronic diseases are more likely to have HBP. The prevalence of diabetes and hyperlipidemia among hypertension were 22.32% and 9.4% among hypertensive patients (Wang et al., 2017), and the rate was even higher among the elderly (Huang et al., 2017).

The prevalence of hypertension has significantly increased with rapid economic development, urbanization, acceleration of population aging, and changes in traditional lifestyle in China. Although many studies have focused on hypertension in China (Hu et al., 2017; Li et al., 2017a; Li et al., 2017b; Lu et al., 2017), still marked ethical and regional differences exist in its prevalence. The rapid and disproportional economic growth in urban and rural China has brought unexpected changes from a public health perspective. A study of hypertension rates and its factors in northeast China presented a higher prevalence in urban areas and a urban-rural disparity in their risk factors (Wang et al., 2018a). However, this investigation cannot be generalized to the entire country. Elderly are the major population who suffer from hypertension the most. Despite China’s economic growth, social progress, improved living standards and accelerated urbanization, there were few cross-sectional studies comparing risk factors of elderly hypertension from regional perspective. The difference in their demographic composition and healthy lifestyles as well as comorbidity would finally contribute to their health status. Also, the previous study mostly focused on difference between urban and rural population without analysing characteristics and behaviours by different genders. To make further implementations of hypertension intervention among male and female elderly from regional perspective, this study aimed to assess regional hypertension prevalence and to explore disparities of its risk factors among urban and rural elderly.

## Materials & Methods

### Data and sample

Data for this study were collected from the China Health and Retirement Longitudinal Study (CHARLS) 2015. A four-stage, stratified, cluster sampling of 25,504 individuals in 28 provinces, 150 counties and 450 villages or communities was drawn by Charles-GIS software. In the first stage, 150 county-level units from 28 provinces were selected to give a mix of urban and rural settings and a wide variation in the level of economic development. All of the dwellings in each selected primary sampling unit were outlined on Google Earth maps using the CHARLS-GIS software package that was specifically designed for the survey. Finally, for the present investigation, 24 of the mapped households in each primary sampling unit were randomly selected (Zhao, John & Yang, 2013). In total 21,097 respondents were interviewed in 2015.

Inclusion criteria for this study included that: (i) subjects who were older than 60 years; (ii) subjects who had provided Blood Pressure (BP) measures. Exclusion criteria included subjects containing missing values for individual characteristics, waist circumference, Body-mass index (BMI), health behaviors and diagnosed chronic diseases. 1,776 participants were selected for analysis of this study.

Respondents were asked to relax and remain seated when BP was measured 3 times at 5-minute intervals using an Omron HEM-7200 Monitor during the day time. Respondents were asked whether they were taking anti-hypertensive medicine. A sample of 6,949 respondents above 60 years old had provided complete data for all study variables related to hypertension risk factors. The original CHARLS was approved by the Ethical Review Committee (IRB) at Peking University (IRB00001052–11015). A “Letter to the Residents” leaflet was sent to the selected households and the arrangements for the survey was permitted by all residents participated. The datasets analysed during the current study are publicly available on http://charls.pku.edu.cn/en/page/data/2015-charls-wave4.

### Measurement

Hypertension was defined as (i) average systolic blood pressure (SBP) ≥ 140 mm Hg or average diastolic blood pressure (DBP) ≥ 90 mm Hg or higher, or (ii) the respondent was currently taking antihypertensive medications. ‘Sociodemographic characteristics included age, sex, educational attainment (no education, primary school, middle school and above), cohabitation status, household income, insurance, central obesity, BMI, health behaviors and diagnosed chronic diseases. The rural or urban administrative registration status is based on the National Bureau of Statistics definition where a PSU is defined as urban if it is located in a city, suburb of a city, a town, suburb of a town, or other special areas where nonfarm employment constitutes at least 70% of the workforce, such as a special econom-tailed). ic zone, state-owned farm enterprise. Living with a partner was defined as living with a spouse or a cohabitant. Household income was categorized into 4 groups based on the 25th, 50th, and 75th percentiles. BMI was defined as weight (kg) divided by height^2^ (m^2^) (<18.5 as low, 18.5–23.9 as normal, 24–27.9 as overweight and ≥ 28 as obesity) (Xu, Lai & Fang, 2016). Central obesity was defined as over 85 cm for males and over 80 cm for females (Chen & Lu, 2004). Health behaviors included physical activity, alcohol intake and smoking. Physical activity was assessed by whether subjects took any light, moderate or vigorous exercise recently. Alcohol intake was assessed by the question “Did you ever drink?” and “do you currently drinking?” Smokers were identified by the question “Did you ever smoke?” and “Do you currently smoking?” Diagnosed chronic diseases included diabetes, dyslipidemia, chronic lung diseases, kidney diseases, digestive diseases, psychiatric problems.

### Statistical analysis

The prevalence was estimated using 95% CIs to show their ranges. The results were expressed in frequencies and percentages. Complex sample plan was created according to stratification, cluster and individual weight without non-response adjusted in this survey for chi-square test and multivariable logistic regression for cluster effect adjust. Basic sociodemographic variables were described by descriptive statistics. Chi-square bivariate tests were performed to examine the group differences (hypertension or not) for all the demographic variables. The association between hypertension and the independent variables was measured by logistic regression. Four separate regression models were run for each gender of the urbanity variable. Results of the regression analysis were presented as adjusted ORs with corresponding 95% CIs. The statistical significance test level was set at 0.05 (two-tailed). All statistical analysis was implemented by using SPSS 13.0 (SPSS Inc., Chicago, IL, USA).

## Results

### Population characteristics

Table 1 shows that the average age of the population was 68 years old. About one third of the population were from urban (34.0%) areas. The male ratio was 50.1%. The majority of the population was living with a spouse or a cohabitant. The rate of illiteracy was 31.9% and 47.2% finished primary school. There were more subjects educated in urban than that in rural areas. Most subjects were insured. The majority of the population were in the normal BMI level (48.9%) and the least belonged to the low level (7.3%). A BMI disparity was found between urban and rural population as the urban population had higher proportion of residents in the high BMI level (40.9% vs 26.2%). Most subjects did light to moderate level physical activity. Subjects in rural did more vigorous activity than urban ones. Most of the population never drank or smoke. The prevalence of drinking was 33.5% and smoking was 28.7%. The urban population also had higher rates of diabetes (16.4% vs 10.0%) and dyslipidemia (30.1% vs 18.1%). The prevalence of chronic lung diseases, psychiatrics, kidney diseases and digestive diseases were respectively 19.0%, 2.6%, 12.2% and 33.2%.

**Table 1:** Sample characteristics and prevalence of hypertension of the participants.

		Total			Urban			Rural		
Variable	Catergory	N (%)	Prevalence (95% CI)	P	N (%)	Prevalence (95% CI)	P	N (%)	Prevalence (95% CI)	P
Region	Urban	604 (34.99)	48.57 (44.49–52.64)							
	Rural	1172 (65.99)	47.16 (44.00–50.32)							
				0.59						
Sex	male	890 (5.112)	47.50 (43.88–51.11)		282 (46.68)	51.75 (45.60–57.90)		608 (51.87)	45.48 (45.60–57.90)	
	female	886 (49.88)	47.79 (44.53–51.05)		322 (53.31)	45.66 (39.74–51.58)		564 (48.12)	49.01 (39.74–51.58)	
				0.87			0.15			0.14
Age	60-69	1233 (69.42)	45.02 (42.13–47.90)		392 (64.96)	46.22 (41.27–51.17)		841 (71.75)	44.43 (41.27–51.17)	
	70-79	460 (25.99)	51.86 (46.98–56.74)		178 (29.47)	52.58 (45.10–60.06)		282 (24.61)	51.43 (45.10–60.06)	
	80-	83 (4.673)	60.65 (49.64–71.67)		34 (5.63)	54.96 (37.97–71.95)		49 (4.19)	64.24 (37.97–71.95)	
				<0.01			0.24			0.01
Living with a partner	yes	1382 (77.81)	46.47 (43.61–49.34)		454 (75.16)	48.15 (43.46–52.84)		928 (79.18)	45.64 (43.46–52.84)	
	no	394 (22.18)	51.60 (46.14–57.06)		150 (24.83)	49.79 (40.69–58.89)		244 (2.82)	52.72 (40.69–58.89)	
				0.12			0.74			0.07
Literacy	Uneducated	567 (31.92)	48.53 (44.41–52.66)		132 (21.85)	46.39 (37.33–55.45)		435 (37.11)	49.15 (37.33–55.45)	
	Primary school	838 (47.18)	48.21 (44.58–51.84)		281 (46.52)	54.01 (47.85–60.18)		557 (47.52)	45.27 (47.85–60.18)	
	Middle school and above	371 (2.889)	45.03 (38.70–51.37)		191 (31.62)	42.01 (33.19–50.83)		180 (15.35)	48.45 (33.19–50.83)	
				0.56			0.06			0.44
^*^Houshold income	poor	446 (25.11)	48.01 (42.80–53.22)		102 (16.88)	49.94 (39.21–60.67)		344 (29.35)	47.48 (39.21–60.67)	
	near poor	477 (26.85)	47.10 (41.98–52.22)		94 (15.56)	46.49 (35.63–57.35)		383 (32.67)	47.25 (35.63–57.35)	
	middle income	443 (24.94)	48.16 (43.25–53.07)		149 (24.66)	51.11 (42.67-59.56)		294 (25.85)	46.60 (42.67–59.56)	
	rich	410 (23.85)	47.35 (41.88–52.83)		259 (42.88)	47.43 (40.54–54.31)		151 (12.88)	47.23 (40.54–54.31)	
				0.99			0.83			1.00
Insured	yes	1632 (91.89)	47.33 (44.82–49.85)		549 (9.89)	47.24 (43.08–51.40)		1083 (92.46)	47.38 (43.08–51.40)	
	no	144 (8.181)	50.95 (41.21–60.69)		55 (9.16)	61.12 (43.82–78.43)		89 (7.593)	44.54 (43.82–78.43)	
				0.47			0.11			0.59
BMI	low	129 (7.263)	32.34 (23.49–41.18)		38 (6.29)	35.59 (16.20–54.99)		91 (7.76)	30.90 (16.20–54.99)	
	normal	869 (48.93)	38.54 (34.93–42.16)		244 (4.40)	38.84 (31.83–45.85)		625 (53.32)	38.43 (31.83–45.85)	
	high	554 (31.19)	56.74 (52.03–61.46)		247 (4.89)	54.24 (46.92–61.57)		307 (26.19)	58.94 (46.92–61.57)	
	obesity	224 (12.61)	70.88 (65.24–76.51)		75 (12.41)	68.99 (59.59–78.40)		149 (12.71)	71.76 (59.59–78.40)	
				<0.01			<0.01			<0.01
Central obesity	yes	699 (39.35)	35.61 (31.72–39.49)		188 (31.12)	35.74 (27.57–43.91)		511 (43.66)	35.56 (27.57–43.91)	
	no	1077 (6.641)	55.59 (52.40–58.79)		416 (68.87)	54.40 (49.60–59.20)		661 (56.39)	56.36 (49.60–59.20)	
				<0.01			<0.01			<0.01
Physical activity	none	225 (12.66)	56.20 (49.00–63.39)		70 (11.58)	62.31 (50.63–74.00)		155 (13.22)	53.70 (50.63–74.00)	
	light	479 (26.97)	52.23 (47.50–56.95)		190 (31.45)	49.68 (42.67–56.70)		289 (24.65)	53.96 (42.67–56.70)	
	moderate	511 (28.77)	47.72 (43.05–52.38)		225 (37.25)	47.65 (40.23–55.06)		286 (24.42)	47.77 (40.23–55.06)	
	vigrous	561 (31.58)	40.14 (36.03–44.25)		119 (19.71)	41.07 (32.65–49.48)		442 (37.71)	39.89 (32.65–49.48)	
				<0.01			0.02			<0.01
Drink	never	944 (53.15)	48.61 (45.28–51.93)		336 (55.62)	48.25 (42.70–53.80)		608 (51.87)	48.80 (42.70–53.80)	
	quit	237 (13.34)	49.03 (42.30–55.77)		83 (13.74)	52.55 (40.25–64.85)		154 (13.13)	47.11 (40.25–64.85)	
	yes	595 (33.52)	45.55 (41.20–49.89)		185 (3.63)	47.34 (39.27–55.42)		410 (34.98)	44.72 (39.27–55.42)	
				0.49			0.75			0.41
Smoke	never	923 (51.97)	47.32 (44.11–50.52)		331 (54.81)	45.05 (39.71–50.38)		592 (5.51)	48.56 (39.71–50.38)	
	quit	343 (19.31)	59.22 (53.48–64.97)		105 (17.38)	65.68 (56.27–75.08)		238 (2.37)	56.14 (56.27–75.08)	
	yes	510 (28.71)	40.18 (35.49–44.87)		168 (27.81)	43.78 (35.57–51.98)		342 (29.18)	38.35 (35.57–51.98)	
				<0.01			<0.01			<0.01
Diabetes	yes	216 (12.16)	64.07 (57.10–71.05)		99 (16.39)	71.74 (62.55–80.92)		117 (9.98)	57.68 (62.55–80.92)	
	no	1560 (87.83)	45.50 (42.81–48.19)		505 (83.69)	44.37 (39.80–48.95)		1055 (9.18)	46.05 (39.80–48.95)	
				<0.01			<0.01			0.02
Dyslipidemia	yes	394 (22.18)	62.92 (57.98–67.87)		182 (3.13)	59.96 (53.26–66.66)		212 (18.88)	65.70 (53.26–66.66)	
	no	1382 (77.81)	43.34 (40.61–46.07)		422 (69.86)	43.46 (38.26–48.66)		960 (81.91)	43.29 (38.26–48.66)	
				<0.01			<0.01			<0.01
Lung diseases	yes	337 (18.97)	48.74 (43.25–54.23)		107 (17.71)	52.68 (43.56–61.80)		230 (19.62)	46.90 (43.56–61.80)	
	no	1439 (81.24)	47.39 (44.69–50.09)		497 (82.28)	47.70 (42.93–52.47)		942 (8.375)	47.22 (42.93–52.47)	
				0.65			0.31			0.92
Psychiatric problem	yes	47 (2.646)	56.88 (40.77–72.99)		18 (2.98)	54.47 (28.30–80.64)		29 (2.47)	58.54 (28.30–80.64)	
	no	1729 (97.35)	47.39 (44.82–49.95)		586 (97.19)	48.37 (44.01–52.73)		1143 (97.52)	46.88 (44.01–52.73)	
				0.26			0.63			0.25
Kidney diseases	yes	216 (12.16)	42.98 (35.90–50.07)		68 (11.25)	47.56 (35.33–59.78)		148 (12.62)	40.96 (35.33–59.78)	
	no	1560 (87.83)	48.29 (45.64–50.95)		536 (88.74)	48.69 (44.28–53.10)		1024 (87.37)	48.08 (44.28–53.10)	
				0.15			0.85			0.08
Digestive diseases	yes	589 (33.16)	43.98 (39.76–48.19)		186 (3.79)	45.39 (37.80–52.98)		403 (34.38)	43.30 (37.80–52.98)	
	no	1187 (66.83)	49.47 (46.43–52.50)		418 (69.25)	50.01 (44.74–55.28)		769 (65.61)	49.17 (44.74–55.28)	
				0.04			0.31			0.05

**Notes.**

* Household income were categorized into 4 groups based on the 25th, 50th, and 75th percentiles-poor (0-1,575 RMB), near poor (1,575-4,504 RMB), middle income (4,504-24,820 RMB), and rich (above 24,820 RMB).

### The urban-rural disparity of prevalence in hypertension

Prevalence of HBP was 47.6% (95% CI [45.2%–50.1%]) in total and it was close between urban and rural population (48.6% vs 47.2%). The prevalence was higher among those who were overweight, overall and central obese, with lower physical activity, smoke quitters, with diabetes and dyslipidemia and among older rural subjects. Table 2 shows the prevalence of HBP stratified by sex in both urban and rural areas. Results of cross-tabulation shows that HBP was more prevalent among both males and females in rural and urban areas who had higher BMI levels or central obesity; among urban males who quit smoke or had diabetes; among urban females who were under middle school education, with low physical activity, quit smoke, with diabetes, dyslipidemia, or psychiatric problems; among rural males who were older, smoke quitters, with diabetes or dyslipidemia; among rural females who had low physical activity, with dyslipidemia or without digestive diseases.

**Table 2:** Prevalence of hypertension across the explanatory variables.

		Urban						Rural					
		Male			Female			Male			Female		
Variable	Catergory	N(%)	Prevalence (95% CI)	P	N(%)	Prevalence (95% CI)	P	N(%)	Prevalence (95% CI)	P	N(%)	Prevalence (95% CI)	P
Age	60-69	173 (61.34)	50.27 (42.74–57.81)		219 (68.12)	42.78 (35.50–50.05)		434 (71.38)	43.12 (38.50–47.75)		407 (72.16)	45.85 (41.15-50.55)	
	70-79	93 (32.97)	52.91 (43.02–62.79)		85 (26.39)	52.24 (41.12–63.35)		148 (24.34)	46.98 (39.41–54.55)		134 (23.75)	56.42 (47.90–64.94)	
	80-	16 (5.67)	61.12 (34.42–87.82)		18 (5.60)	48.93 (28.81–69.06)		26 (4.276)	70.53 (53.48–87.58)		23 (4.78)	56.76 (36.30–77.22)	
				0.56			0.23			0.01			0.05
Living with a partner	yes	238 (84.39)	50.71 (44.40–57.02)		216 (67.87)	45.15 (37.65–52.64)		510 (83.88)	44.84 (40.32–49.37)		418 (74.11)	46.62 (41.81-51.43)	
	no	44 (15.62)	57.35 (41.79–72.91)		106 (32.91)	46.64 (35.77–57.50)		98 (16.11)	48.50 (36.53–60.47)		146 (25.88)	55.82 (47.84–63.80)	
				0.34			0.81			0.57			0.05
Literacy	uneducated	25 (8.87)	40.51 (20.75–60.27)		107 (33.22)	47.76 (36.71–58.82)		106 (17.43)	44.59 (34.73–54.45)		329 (58.33)	50.55 (45.27–55.83)	
	primary school	148 (52.48)	56.75 (48.41–65.09)		133 (41.34)	50.99 (42.25–59.73)		351 (57.73)	43.50 (37.64–49.36)		206 (36.52)	48.41 (40.94–55.88)	
	middle school and above	109 (38.65)	47.48 (36.44–58.52)		82 (25.46)	34.32 (23.45–45.19)		151 (24.83)	50.90 (41.94–59.86)		29 (5.141)	35.22 (16.34–54.10)	
				0.15			0.03			0.35			0.33
Houshold income	poor	45 (15.95)	53.96 (39.37–68.55)		57 (17.71)	46.75 (30.92–62.58)		169 (27.79)	45.16 (37.35–52.97)		175 (31.28)	49.82 (41.80–57.84)	
	near poor	42 (14.89)	44.43 (32.51–56.36)		52 (16.14)	48.17 (32.35–63.98)		206 (33.88)	46.48 (38.60–54.36)		177 (31.38)	48.14 (40.44–55.85)	
	middle income	67 (23.75)	56.58 (44.33–68.84)		82 (25.46)	46.57 (35.00–58.14)		153 (25.16)	43.76 (36.21–51.31)		141 (25.00)	49.75 (41.59–57.91)	
	rich	128 (45.39)	50.96 (40.46–61.45)		131 (4.68)	43.73 (35.14–52.32)		80 (13.15)	46.60 (34.98–58.23)		71 (12.58)	47.94 (36.86–59.03)	
				0.46			0.90			0.96			0.98
Insured	yes	267 (94.68)	49.45 (42.91–55.98)		282 (87.57)	45.16 (39.43–50.90)		570 (93.75)	45.15 (40.82–49.48)		513 (9.957)	49.91 (45.90–53.91)	
	no	15 (5.319)	77.36 (53.07–101.6)		40 (12.42)	49.76 (31.24–68.29)		38 (6.25)	50.03 (35.01–65.06)		51 (9.43)	40.21 (24.23–56.19)	
				0.04			0.56			0.50			0.19
BMI	low	20 (7.92)	27.07 (7.151–47.00)		18 (5.60)	42.41 (14.52–70.30)		40 (6.58)	31.24 (15.55–46.92)		51 (9.43)	30.65 (19.01–42.30)	
	normal	137 (48.58)	43.98 (34.64–53.32)		107 (33.22)	32.17 (23.05–41.28)		368 (6.526)	37.52 (32.18–42.86)		257 (45.56)	39.78 (33.48–46.07)	
	high	103 (36.52)	60.06 (48.57–71.54)		144 (44.72)	49.60 (40.78–58.42)		140 (23.26)	61.63 (52.89–70.38)		167 (29.69)	56.66 (48.85–64.46)	
	obesity	22 (7.81)	76.79 (61.41–92.16)		53 (16.45)	65.41 (52.33–78.48)		60 (9.87)	68.28 (57.38–79.19)		89 (15.78)	74.17 (64.34–83.99)	
				<0.01			<0.01			<0.01			<0.01
Central obesity	yes	119 (42.19)	38.92 (28.97–48.88)		69 (21.42)	30.49 (17.82–43.15)		341 (56.85)	35.82 (30.26–41.38)		170 (3.141)	35.02 (28.14–41.90)	
	no	163 (57.81)	60.59 (52.62–68.56)		253 (78.57)	50.08 (43.43–56.73)		267 (43.91)	58.08 (51.19–64.97)		394 (69.85)	55.18 (50.28–60.08)	
				<0.01			<0.01			<0.01			<0.01
Physical activity	none	36 (12.76)	50.99 (32.16–69.83)		34 (1.559)	75.52 (59.09–91.94)		79 (12.99)	50.83 (37.34–64.33)		76 (13.47)	57.04 (44.73–69.35)	
	light	89 (31.56)	47.60 (37.09–58.11)		101 (31.36)	51.50 (41.92–61.09)		150 (24.67)	53.07 (44.33–61.81)		139 (24.64)	54.91 (45.52–64.30)	
	moderate	86 (3.50)	56.42 (43.31–69.54)		139 (43.16)	41.84 (32.58–51.10)		122 (2.657)	45.29 (34.81–55.77)		164 (29.78)	49.65 (41.97–57.33)	
	vigrous	71 (25.17)	51.47 (40.75–62.19)		48 (14.96)	24.25 (14.08–34.42)		257 (42.26)	39.29 (33.24–45.34)		185 (32.81)	40.72 (33.52–47.93)	
				0.64			<0.01			0.08			0.04
Drink	never	72 (25.53)	55.27 (42.83–67.70)		264 (81.98)	46.12 (39.49–52.74)		181 (29.76)	44.99 (36.88–53.10)		427 (75.79)	50.47 (45.88–55.05)	
	quit	59 (2.92)	53.41 (38.68–68.14)		24 (7.45)	50.55 (29.60–71.49)		106 (17.43)	45.15 (35.18–55.12)		48 (8.52)	51.19 (36.99–65.40)	
	yes	151 (53.54)	49.29 (40.37–58.22)		34 (1.56)	38.45 (22.20–54.71)		321 (52.79)	45.86 (40.23–51.49)		89 (15.78)	40.34 (28.45–52.23)	
				0.69			0.53			0.97			0.23
Smoke	never	52 (18.43)	44.86 (32.08–57.65)		279 (86.64)	45.08 (39.09–51.07)		93 (15.29)	44.83 (33.10–56.56)		499 (88.47)	49.30 (44.97–53.63)	
	quit	88 (31.25)	65.01 (54.85–75.17)		17 (5.28)	69.18 (43.14–95.21)		212 (34.86)	58.02 (50.77–65.26)		26 (4.70)	40.67 (21.21–60.14)	
	yes	142 (5.35)	45.31 (35.86–54.77)		26 (8.745)	35.92 (19.14–52.71)		303 (49.83)	36.72 (30.67–42.76)		39 (6.91)	50.86 (36.17–65.55)	
				<0.01			0.03			<0.01			0.73
Diabetes	yes	37 (13.12)	69.79 (54.72–84.86)		62 (19.25)	72.98 (61.14–84.82)		49 (8.592)	63.44 (48.80–78.09)		68 (12.56)	53.50 (41.72–65.28)	
	no	245 (86.87)	49.17 (42.57–55.77)		260 (8.75)	39.70 (33.15–46.25)		559 (91.94)	43.98 (39.40–48.56)		496 (87.94)	48.42 (44.18–52.66)	
				0.01			<0.01			0.02			0.35
Dyslipidemia	yes	78 (27.65)	58.34 (47.51–69.18)		104 (32.29)	61.31 (50.26–72.36)		108 (17.76)	66.43 (57.23–75.63)		104 (18.43)	64.90 (54.90–74.90)	
	no	204 (72.34)	48.99 (41.66–56.32)		218 (67.71)	38.20 (31.33–45.07)		500 (82.23)	41.13 (36.34–45.93)		460 (81.56)	45.67 (41.40–49.94)	
				0.06			<0.01			<0.01			<0.01
Lung diseases	yes	62 (21.98)	53.73 (41.02–66.44)		45 (13.97)	51.08 (34.83–67.33)		134 (22.39)	46.70 (37.51–55.88)		96 (17.21)	47.20 (37.78–56.62)	
	no	220 (78.14)	51.19 (43.90–58.48)		277 (86.24)	44.84 (38.55–51.13)		474 (77.96)	45.13 (40.49–49.77)		468 (82.97)	49.36 (45.10–53.62)	
				0.69			0.43			0.73			0.63
Psychiatric problem	yes	8 (2.84)	30.37 (-1.75-62.51)		10 (3.16)	72.86 (46.70–99.02)		10 (1.64)	65.38 (33.74–97.02)		19 (3.37)	53.59 (30.20–76.98)	
	no	274 (97.16)	52.39 (45.96–58.81)		312 (96.89)	44.68 (38.52–50.84)		598 (98.35)	45.08 (40.77–49.39)		545 (96.63)	48.87 (44.87–52.86)	
				0.22			<0.01			0.21			0.70
Kidney diseases	yes	36 (12.76)	45.80 (30.56–61.05)		32 (9.94)	49.44 (30.19–68.70)		91 (14.96)	41.65 (29.88–53.42)		57 (1.16)	39.84 (26.05–53.62)	
	no	246 (87.23)	52.55 (46.11–58.99)		290 (9.62)	45.23 (39.02–51.45)		517 (85.32)	46.17 (41.50–50.84)		507 (89.89)	50.06 (45.89–54.23)	
				0.31			0.65			0.38			0.13
Digestive diseases	yes	78 (27.65)	47.09 (35.64–58.54)		108 (33.54)	44.17 (33.13–55.21)		175 (28.78)	44.77 (36.96–52.57)		228 (4.425)	42.13 (35.74–48.52)	
	no	204 (72.34)	53.51 (45.43–61.59)		214 (66.45)	46.46 (39.93–52.99)		433 (71.21)	45.77 (40.79–50.75)		336 (59.57)	53.59 (48.64–58.54)	
				0.34			0.68			0.78			0.01

### The urban-rural disparity of risk factors associated with hypertension

Table 3 shows results of regression analysis of impact factors associated with HBP prevalence. Age, literacy, BMI level, physical activity, smoking, and having comorbidities of diabetes and dyslipidemia were associated with HBP.

**Table 3:** Associated risk factors of hypertension in rural and urban areas.

		**Urban**		**Rual**	
		**Male**	**Female**	**Male**	**Female**
**Variable**	**Category**	**OR(95% CI)**	**OR(95% CI)**	**OR(95% CI)**	**OR(95% CI)**
Age	70-79	1.124 (0.680–1.857)	1.074 (0.665–1.734)	1.343 (0.941–1.916)	^**^1.934 (1.282–2.917)
	80-	1.551 (0.388–6.204)	0.900 (0.365–2.220)	^**^3.695 (1.688–8.091)	2.175 (0.825–5.734)
	60-69	1	1	1	1
Living with a partner	no	1.994 (0.898–4.431)	0.945 (0.460–1.944)	1.255 (0.789–1.997)	1.499 (0.930–2.414)
	yes	1	1	1	1
Literacy	uneducated	0.605 (0.230–1.591)	0.875 (0.494–1.549)	1.054 (0.670–1.658)	1.141 (0.758–1.719)
	middle school and above	^*^0.475 (0.275–0.819)	^*^0.326 (0.144–0.738)	1.254 (0.784–2.006)	0.558 (0.216–1.438)
	primary school	1	1	1	1
Household income	poor	0.696 (0.324–1.497)	1.246 (0.571–2.720)	1.169 (0.739–1.849)	1.002 (0.606–1.656)
	near poor	0.671 (0.341–1.319)	1.692 (0.722–3.964)	1.114 (0.720–1.723)	0.949 (0.565–1.595)
	rich	0.602 (0.338–1.074)	1.217 (0.710–2.085)	1.031 (0.586–1.814)	0.890 (0.451–1.755)
	middle income	1	1	1	1
Insured	no	2.460 (0.661–9.153)	0.856 (0.416–1.761)	1.378 (0.748–2.536)	0.543 (0.259–1.140)
	yes	1	1	1	1
BMI	low	0.400 (0.166–0.961)	2.115 (0.462–9.685)	0.736 (0.308–1.760)	0.607 (0.336–1.098)
	high	1.572 (0.763–3.236)	1.626 (0.898–2.946)	^**^2.135 (1.272–3.582)	^**^1.831 (1.187–2.825)
	obesity	^*^3.319 (1.154–9.546)	2.197 (1.088–4.439)	^**^2.398 (1.374–4.184)	^**^3.707 (1.933–7.109)
	normal	1	1	1	1
Central obesity	no	1.556 (0.753–3.216)	1.831 (0.742–4.515)	1.425 (0.874–2.324)	1.409 (0.917–2.165)
	yes	1	1	1	1
Physical activity	light	0.758 (0.319–1.802)	^**^0.226 (0.105–0.486)	0.978 (0.521–1.836)	1.138 (0.614–2.107)
	moderate	1.219 (0.496–2.996)	^**^0.185 (0.076–0.451)	0.901 (0.465–1.743)	0.854 (0.450–1.620)
	vigorous	1.044 (0.451–2.414)	^**^0.078 (0.034–0.180)	0.668 (0.363–1.230)	0.733 (0.387–1.387)
	none	1	1	1	1
Drink	quit	0.978 (0.381–2.512)	1.012 (0.430–2.378)	0.785 (0.444–1.389)	0.860 (0.423–1.748)
	yes	0.871 (0.459–1.652)	0.842 (0.439–1.615)	1.160 (0.745–1.805)	0.640 (0.376–1.087)
	never	1	1	1	1
Smoke	quit	^*^2.432 (1.194–4.950)	2.314 (1.112–4.813)	1.133 (0.582–2.208)	0.736 (0.316-1.712)
	yes	1.420 (0.800–2.520)	0.776 (0.360–1.673)	0.760 (0.423–1.364)	1.571 (0.820–3.009)
	never	1	1	1	1
Diabetes	yes	^*^2.836 (1.072–7.501)	^**^2.960 (1.560–5.619)	1.266 (0.654–2.451)	0.721 (0.396–1.313)
	no	1	**1**	1	1
Dyslipidemia	yes	1.216 (0.656–2.254)	^*^2.148 (1.168–3.949)	^**^2.158 (1.305–3.568)	1.685 (0.922-3.078)
	no	1	**1**	1	1
Lung diseases	yes	1.139 (0.655–1.982)	0.950 (0.459–1.967)	1.103 (0.711–1.711)	1.058 (0.676–1.655)
	no	1	1	1	1
Kidney diseases	yes	0.524 (0.250–1.099)	1.036 (0.463–2.317)	0.686 (0.422–1.113)	0.718 (0.393–1.310)
	no	1	1	1	1
Digestive diseases	yes	0.695 (0.382–1.265)	0.684 (0.383–1.222)	1.150 (0.783–1.688)	0.676 (0.465–0.981)
	no	1	1	1	1
Psychiatric problem	yes	0.241 (0.043–1.347)	1.973 (0.341–11.41)	1.470 (0.384–5.626)	1.486 (0.618–3.573)
	no	1	1	1	1

**Notes.**

OR Odds Ratio 95% CI 95% Confidence interval for odds ratio

* *P* < 0.05.

** *P* < 0.01.

In urban areas, HBP prevalence was associated with the education of males and females. Males and females who had middle school education and above had 62.4% (OR = 0.376, 95% CI [0.275–0.819]) and 67.4% (OR = 0.326, 95% CI [0.144–0.738]) lower odds of HBP. Urban males who were obese had 2.319 times (OR = 3.319, 95% CI [1.154–9.546]) higher odds of HBP, those who quit smoking had 1.432 times higher odds (OR = 2.432, 95% CI [1.194–4.950]) of HBP, and those who had diabetes had 1.836 times higher odds (OR = 2.836, 95% CI [1.072–7.501]) of HBP. Urban females had light, moderate, and vigorous physical activity had respectively 87.4% (OR = 0.226 95% CI [0.105–0.486]), 91.5% (OR = 0.185, 95% CI [0.076–0.451]), and 92.2% (OR = 0.078 95% CI [0.034–0.180]) lower odds of HBP than those who never exercise. Urban females who had diabetes and dyslipidemia had respectively 1.96 times (OR = 2.960, 95% CI [1.560–5.619]) and 1.148 times (OR = 2.148, 95% CI [1.168–3.949]) higher odd of HBP.

In rural areas, males who were above 80 years old had 2.695 times higher odds (OR = 3.695, 95% CI [1.688–8.091]) of HBP than those who were 60 to 69. Rural females who were 70 to 79 had 93.4% times higher odds (OR = 1.934, 95% CI [1.282–2.917]) of HBP than those who were 60 to 69. Among rural males and females, those who were overweight had respectively 1.135 times (OR = 2.135 95% CI [1.272–3.582]) and 83.1% times (OR = 1.831, 95% CI [1.187–2.825]) higher odds of HBP, and those who were obese had respectively 1.398 times (OR = 2.398, 95% CI [1.374–4.184]) and 1.707 times (OR = 3.707 95% CI [1.933–7.109]) higher odds of HBP than those had normal weight. Also among rural males, those who had dyslipidemia had 1.158 times higher odds (OR = 2.158, 95% CI [1.305–3.568]) of HBP than others.

## Discussion

Based on the nationally representative data from CHARLS, this study explored the disparity of prevalence and risk factors of hypertension among the elderly in China. Our results showed that the prevalence of hypertension was close in urban and rural, which indicated a slightly higher prevalence of HBP in urban areas without statistical significance. Factors associated with HBP were different between urban and rural areas. In urban areas, hypertension was significantly associated with literacy and diabetes in both genders, high BMI level and smoke quitters in males, and physical activity and dyslipidemia in females. In rural areas, hypertension was significantly associated with older age, higher BMI level in both males and females, and dyslipidemia in males.

Results indicated that HBP is highly prevalent in Chinese elderly. Prevalence among the elderly was comparable to a national study in 60–79 years old (48.5% vs 50.1%) (Lu et al., 2017) and other investigations in Southern China (Xu, Lai & Fang, 2016; Zhou et al., 2018). However, it was lower than that resulted in a national survey (58.9% in 2012) as well as studies in the Northern areas (Yang et al., 2016; Zhang et al., 2017). The prevalence of hypertension of elderly reported in our study was less than some developed-countries such as Korea (49.7%–64.5%) (Kang et al., 2019) and Japan (57.3%–79.9%) in Asia, as well as other high-income countries such as United States(61.7%) (Fang et al., 2018) and German (56.0%–62.6%) (Labeit et al., 2012). However, it exceeded other middle-income-countries such as Malaysia (Abdul-Razak et al., 2016) and Thailand (Aekplakorn et al., 2012).

The prevalence of hypertension was close in urban and rural areas among the elderly. The prevalence gap between urban and rural areas has gradually narrowed. In 2010, a study of CHARLS found prevalence of urban and rural areas were, respectively, 44.3% and 55.7% (Yin et al., 2016). This finding was consistent with studies reported that seldom disparity (Wang et al., 2018b; Zhou et al., 2018) comparing urban and rural areas. However the higher prevalence in rural was found in a large population screening study in overall (Lu et al., 2017) as well as northeastern part of China (Wang et al., 2018a), while HBP was more prevalent in urban areas in the national survey and national study of prevalence in hypertension (Yang et al., 2017). The results were also found in research from Central (Chen & Yuan, 2018), and Southern China (Hu et al., 2017). It may be caused by sample selection of a certain area. The result is consistent with international urban-rural disparity in hypertension prevalence. The urban-rural disparity of prevalence in hypertension was smaller in upper-middle-income countries (Argentina, Brazil, Malaysia, et al.) (48.5% vs. 50.9%) and lower-middle-income countries (China, Colombia, Iran) (39.9% vs. 39.8%) than high-income (Canada, Sweden, United Arab Emirates) (39.2% vs. 44.6%) and low income countries (Bangladesh, India, Pakistan, Zimbabwe) (38.7% vs. 26.4%) (Chow et al., 2013).

Common risk factors for hypertension included individual characteristics, social economic status, behavioral health, and comorbidities, etc. Due to the significant differences in culture, social background and economy, the hypertension prevalence and its risk factors in urban and rural areas should be different. We designed this study based on national data in China. We also compared differences of risk factors by sex because different health behavior may occur between males and females. Our study showed that in urban areas, obesity and smoking were risk factors for males. Rapid increases in the rates of obesity and overweight are widely documented from urban and rural areas in the poorest countries of sub-Saharan Africa and South Asia to populations in countries with higher income levels (Popkin, Adair & Ng, 2012). Mean BMI in Chinese adults increased from 22.7 kg/m^2^ in 2004 to 23.7 kg/m^2^ (23.6–23.9) in 2010 (Jiang et al., 2015). A study indicated that faster BMI growth rates in the high-social economic status males than females in China (Wang et al., 2015). Urban females who usually have a higher level of education or higher family income are the low risk group for obesity (Wang et al., 2012). A study showed that in contrast to males and rural females, living standard improvement had a negative effect on weight for urban females. It means that urban females pay more attention on physical activity and weights than others (Zhao & Zheng, 2019). There are usually much more males who smoke than females (Li et al., 2016b). A study showed Chinese men smoke more than a third of the world’s cigarettes, following a large increase first in urban then rural usage (Chen et al., 2015). Those who come about health problems are more likely to tend to quit smoke. Above middle school education level was a protective factor of HBP for both urban males and females. Lower levels of education may lead to unhealthy lifestyles and lack of sufficient knowledge to prevent hypertension. A similar finding was shown in a study in Jilin (Wang et al., 2018a). As there is more access to better health service, more people who have high level of education are attracted to urban areas. Having comorbidity of diabetes was risk factor of HBP for both urban males and females, because living in urban area was positively associated with metabolic syndrome risk (Li et al., 2016a; Zuo et al., 2009). The blood lipid profile was less favorable in the urban area. These pronounced urban-rural differences may be related to urbanization, the associated nutrition transition and to access to health care (Pan et al., 2016).

In rural areas, older age and high BMI level were common risk factors for both males and females. Older age was positively associated with rural patients, possibly because the allocation of health resources is uneven between urban and rural areas and the provision of healthcare service is inadequate in rural areas in China (Dai, 2015). Hypertensive patients in urban areas have more access to health service in higher level hospitals which may weaken the relationship of HBP with aging. Research of recent years indicated rural Chinese not only had higher initial BMI status, but also their BMI increased more than urban Chinese. With the rapid development of economy and urbanization in rural areas, rural regions have remarkable changes in lifestyle compared with urban regions (Huang et al., 2018).

Some limitations of this study should be considered. First, our study was a cross-sectional study, which restricted the interpretation of observed associations in terms of cause and effect. Longitude research is required for further investigation of these findings. Second, hypertension diagnosis was not done by 24-hour ambulatory blood pressure monitoring, (O’Brien et al., 2000) which could be the best diagnosis way but would take longer time and not be cost-inefficient in a national survey. In addition, hypertension was associated with multiple risk factors. Most of the risk factors but not all of them were included in this study, such as salt intake which was not investigated in this survey.

## Conclusions

Our findings indicated that hypertension is highly prevalent in the elderly population in China. The prevalence are about the same among urban and rural residents, but their risk factors vary from each other. In urban, hypertension was significantly associated with literacy and diabetes in both genders, high BMI level and smoke quitters in males, and physical activity and dyslipidemia in females. In rural areas, hypertension was significantly associated with older age, higher BMI level in both males and females, and dyslipidemia in males. These findings will provide the baseline information to develop more effective approaches to enhance current prevention and control management of hypertension. In this regard, we suggest taking the disparity in fields into consideration when implementing elderly hypertension intervention.

##  Supplemental Information

10.7717/peerj.8015/supp-1 Supplemental Information 1 HBP prevalence and related risk factors

10.7717/peerj.8015/supp-2 Supplemental Information 2 The complex sample plan for logistic regression model

## Additional Information and Declarations

### Data Availability

The following information was supplied regarding data availability: The raw data is in the Supplemental File. The datasets analyzed in this study are publicly available at the China Health and Retirement Longitudinal Study (CHARLS) site: http://charls.pku.edu.cn/en/page/data/2015-charls-wave4.
